# Juvenile ossifying fibroma of the maxillofacial region: analysis of clinico-pathological features and management

**DOI:** 10.4317/medoral.24592

**Published:** 2021-06-20

**Authors:** Fadi Titinchi

**Affiliations:** 1 partment of Maxillo-Facial and Oral Surgery, Faculty of Dentistry and WHO Collaborating Centre, University of the Western Cape

## Abstract

**Background:**

The diagnosis and management of juvenile ossifying fibroma (JOF) remains a highly debated topic with paucity of studies with long-term follow-up, hence the aim of this study was to report on the clinico-pathological features and management of these neoplasms.

**Material and Methods:**

A retrospective analysis was performed on all histopathologically confirmed JOF presenting at two tertiary hospitals in Cape Town, South Africa over a period of 39 years. Clinical, demographic, histopathological and radiological features were analyzed. Surgical methods were documented and a minimum post-operative follow-up of 12 months was a prerequisite.

**Results:**

Seventeen patients met the inclusion criteria and were included in this study. Overall, the ages of patients ranged from 3–31 years (mean= 13 years) with male to female ratio of 1.8:1. The ages of patients diagnosed with Trabecular JOF were significantly younger than patients with Psammomatoid JOF (*P* = 0.01). The majority of patients presented with marked swelling (88.2%). Interestingly, most neoplasms occurred in the mandible (76.5%) with all Psammomatoid JOF uncharacteristically occurring in the mandible. There was only one case of Trabecular JOF occurring in the sinonasal area. Most neoplasms appeared as unilocular (76.5%) and well-defined (82.4%) with mixed radio-density (70.6%) on radiographs and computed tomography. Curettage with peripheral ostectomy was shown to be the least invasive method with an accepTable recurrence rate (10%). Six lesions underwent resection without any recurrences however caused high morbidity to these young patients.

**Conclusions:**

The high number of lesions occurring in the mandible for both variants of JOF demonstrates that site should not be a major determining factor in the diagnosis of JOF. Moreover, curettage with peripheral ostectomy should be used as the first line of management to minimize morbidity to the patient and that resection should be reserved for large and recurrent lesions.

** Key words:**Juvenile ossifying fibroma, juvenile trabecular ossifying fibroma, juvenile psammomatoid ossifying fibroma, fibro-osseous lesions, maxillofacial region, multi-centre study.

## Introduction

Juvenile ossifying fibroma (JOF) is a subtype of ossifying fibroma (OF) that usually occurs within the maxillofacial region of children and young adults (1). It is an uncommon and debated neoplasm that is differentiated from its adult variant on the basis of age, site, behaviour and microscopic features (2). JOF is divided into two distinct categories: the trabecular and psammomatoid types. Trabecular JOF (JTOF) is identified by the occurrence of trabeculae and fibrillar osteoid and woven bone. The psammomatoid type (JPOF) is identified by the presence of small uniform spherical ossicles that mimic psammoma bodies (3). JTOF affects the maxilla more frequently than the mandible and may display signs of erosion and invasion of the adjacent structures accompanied by rapid enlargement while JPOF primarily involves the sinonasal regions (4).

The management and prognosis of JOF is uncertain. In some cases, it may occur with minimal symptoms, while in other cases, especially in younger patients, it may present with local aggressive behaviour. Therefore, due to the aggressive nature of these tumours with the high recurrence rate, some authors advocate that these locally aggressive neoplasms be treated with surgical resection rather than conservative curettage (1,5).

The clinico-pathological features of JOF differ widely in the literature with few large studies available. Moreover, there are no management protocols based on long-term follow-up; hence the aim of this study was to report on the diagnostic features and management of these neoplasms.

## Material and Methods

A retrospective analysis was performed on all histopathologically confirmed JOF presenting at two tertiary referral hospitals in Cape Town, South Africa from January 1980 to December 2019. This study followed the Declaration of Helsinki on medical protocol and ethics and the regional Ethical Review Board of the University of the Western Cape approved the study.

The inclusion criteria included all records of patients diagnosed with JOF in the maxillofacial region including sinonasal areas with detailed demographic data, clinical features, radiographs and surgical treatment. Each patient’s record included at least one panoramic radiograph as well as any advanced imaging modalities such as computed tomography (CT) when available. CT was performed only in cases where the lesion was extensive and involved areas difficult to assess with conventional radiographs such as the paranasal sinuses. All lesions were histopathologically confirmed by an Oral Pathologist based on the final surgical specimen prior to inclusion in the study. Age and site were not included as part of the diagnostic criteria for diagnosis of JOF.

Records were excluded from the study when the data were incomplete and the histopathological diagnosis was inconclusive. Records were also excluded when the surgical method used to manage the lesion was not described in detail or if the patient did not return for post-operative follow-up to detect any recurrences or complications. The minimum post-operative follow-up period was 12 months.

Each patient’s age, gender and ethnicity were recorded. The clinical features of each neoplasm was recorded as well as the radiological features including site, demarcation, density, size, shape, locularity and its effect on adjacent anatomical structures. The histopathological characteristics were also noted. Surgical management and post-operative follow-up were analysed. All records and radiographs were assessed by one observer (author) and under the same conditions.

The location of the lesion was categorized into different regions in the mandible and maxilla. The anterior region of the mandible extended from the mandibular left canine to right canine. The posterior region of the mandible extended from canine to the angle of the mandible. The anterior region of the maxilla extended from the left maxillary canine to right canine while the posterior region of the maxilla extended from canine to the maxillary tuberosity.

The size of the lesion was measured in centimetres along the widest diameter of the lesion from one border to the opposite border. Radio-density was classified as either radio-lucent, radio-opaque and mixed (radio-lucent with areas of radio-opacity). Lesions were further classified as either unilocular in appearance whereby only one compartment is present or multilocular whereby the lesion appears to be formed of many adjacent compartments within the bone. External root resorption of secondary dentition was assessed on panoramic radiographs for flattening of the apical part of roots of teeth associated with the lesion.

Excel worksheet was used to collect the data. Data analysis was performed using Epi Info® 2000 (Centres for Disease Control and Prevention, Bethesda, MD) by Fisher’s exact test or Mann-Whitney test to compare the findings and to correlate these findings with different parameters. Statistical significance was set at *P* < 0.05.

## Results

- Demographic features

A total of 21 patients were diagnosed with JOF over a period of 39 years. Only 17 patients were included in the study as the other four patients did not meet the inclusion criteria. Overall, the ages of patients ranged from 3-31 years (mean= 13 years) while the ages of patients diagnosed with JTOF were significantly younger than patients with JPOF (*P* = 0.01). Males were more commonly affected than females (M:F ratio = 1.8:1). The majority of patients were of mixed race (n=9; 53%) and African descent (n=8; 47%) while patients of Caucasian and Indian ethnicity were not affected in this sample. Detailed summary of demographic findings for each variant of JOF is shown in Table 1.

- Clinical features

Rapid expansion of the affected facial bones is one of the main identifying features of JOF. The majority of patients presented with marked swelling and facial asymmetry (88.2%) while only two patients (11.8%) complained of pain. The period from initially noticing symptoms to presentation is relatively rapid (mean= 1.9 months) with no statistically significant difference between the two variants of JOF (*P* > 0.05). No patient reported paraesthesia of branches of the trigeminal nerve.

Interestingly, most JOF lesions occurred in the mandible (76.5%) with all JPOF uncharacteristically occurring in the mandible. There was only one case of JTOF occurring in the sinonasal area. The posterior regions of the jaws were more frequently affected.

**Table 1:**
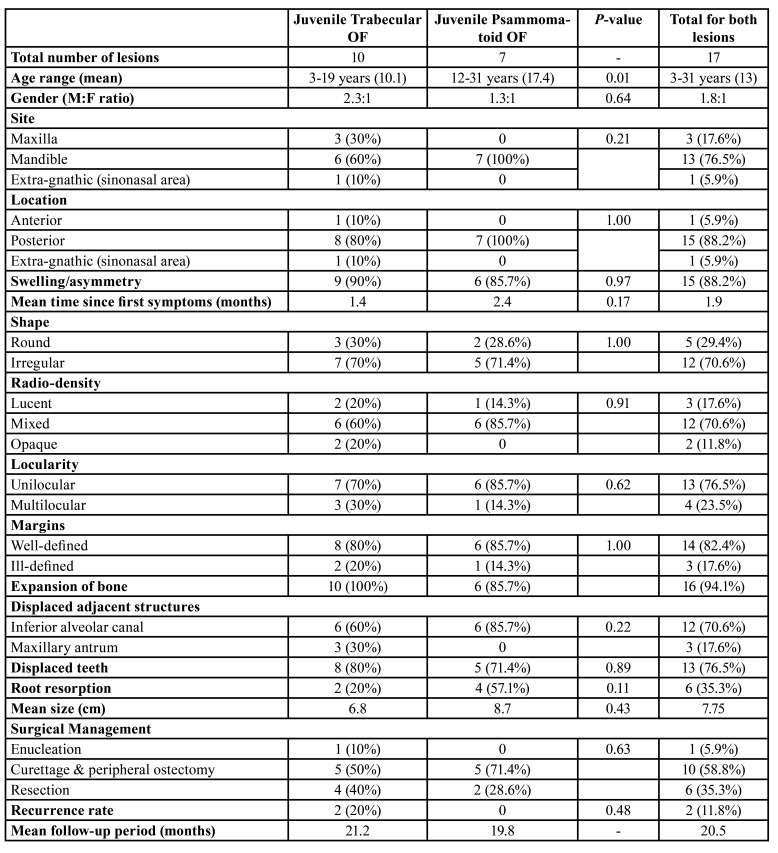
Summary of clinico-pathological features and management of JTOF and JPOF.

- Radiological features

**Figure 1:**
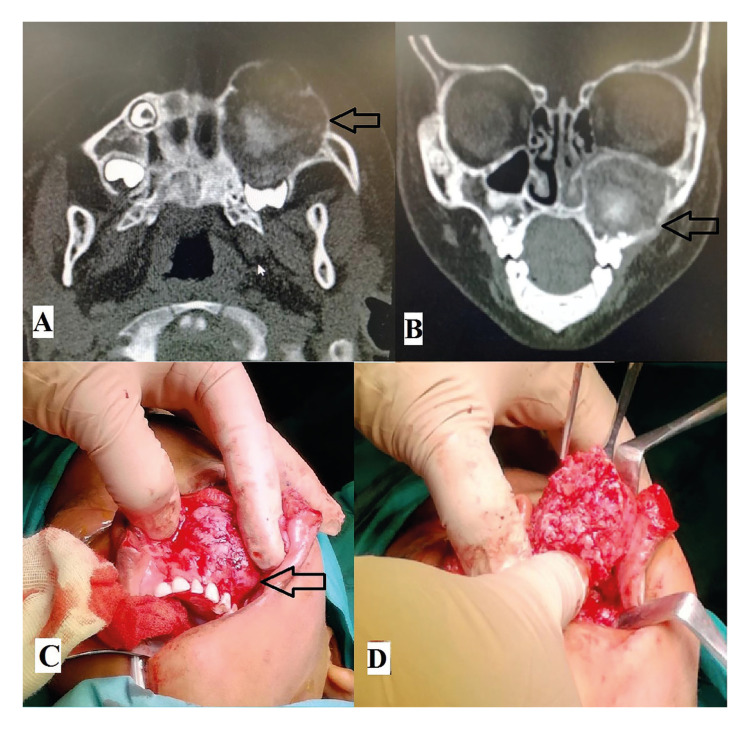
A: Axial CT showing mixed density JTOF lesion with central radio-opacity. B: Coronal CT of same patient showing extension of tumour into maxillary antrum and nasal cavity. C: Intra-operative exposure of neoplasm via Weber-Ferguson incision. D: Curettage of the lesion which was un-encapsulated but well demarcated.

**Figure 2:**
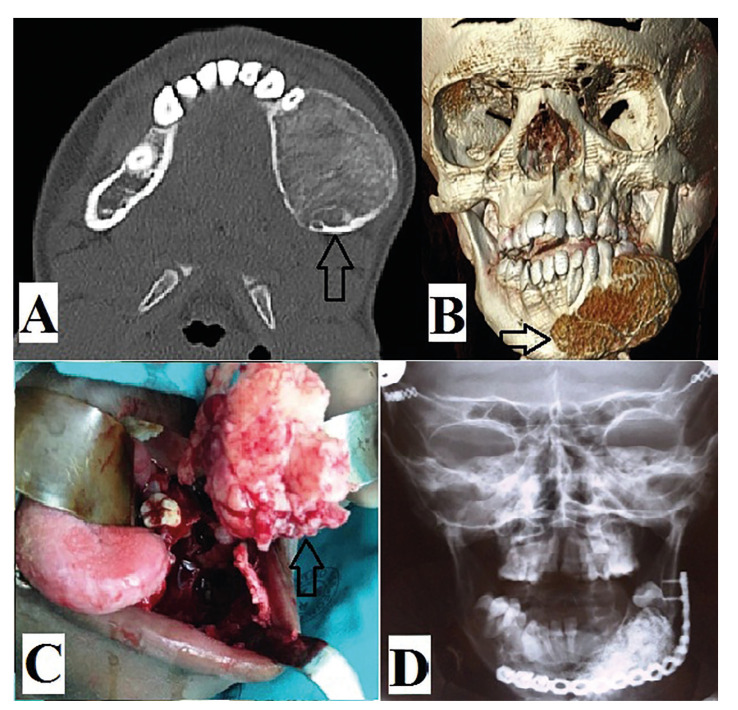
A: Axial CT of JPOF in the posterior mandible showing well-defined mixed density lesion. B: 3D reconstruction of CT showing destruction of mandibular cortex. C: Intra-operative photograph post curettage of lesion. D: Post-operative posterio-anterior radiograph of the mandible following reconstruction of the defect.

Due to their aggressive behaviour, most JOF neoplasms appeared as irregular on panoramic radiographs (70.6%) with few lesions presenting with ill-defined margins (17.6%). Most lesions appeared mixed in radio-density (Fig. 1) while 2 JTOF lesions in the maxilla appeared completely radio-opaque mimicking fibrous dysplasia. Most JOF neoplasms were unilocular in appearance. Mandibular JTOF lesions were more likely to appear as multilocular on conventional radiographs as compared to JPOF; however this finding was not statistically significant (*P* > 0.05). There was marked expansion of the involved bones in nearly all neoplasms as expected (Fig. 2).

In most instances, JOF caused displacement of the inferior alveolar canal or maxillary antrum. Displacement of teeth was a common feature while root resorption was less frequent (35.3%). The difference in size between JTOF and JPOF was however not statistically significant (*P* > 0.05).

- Histopathological features

**Figure 3:**
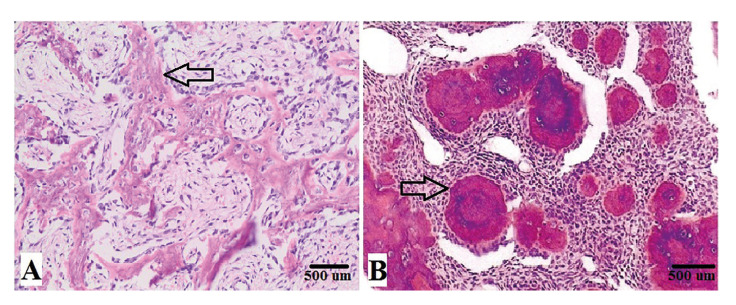
A: Hematoxylin and eosin stained slide showing typical features of JTOF with osteoid formation directly from the hypercellular fibrous stroma. B: Hematoxylin and eosin stained slide showing typical features of JPOF with proliferation of fibroblastic cells and presence of numerous scattered small round psammomatoid calcifications.

All lesions underwent incisional biopsy and histopathological confirmation of diagnosis prior to definitive management. Both lesions were un-encapsulated but were well-delineated from the surrounding uninvolved bone. JTOF showed hypercellular stroma with osteoid developing directly from the fibrous stroma resulting in immature bony trabeculae without osteoblastic rimming (Fig. 3). JPOF also showed fibrous stroma with distinct concentric lamellated and spherical ossicles (Fig. 3). Two cases of JTOF showed associated aneurysmal bone cysts while no lesion showed signs of malignant transformation.

- Management and recurrence

**Figure 4:**
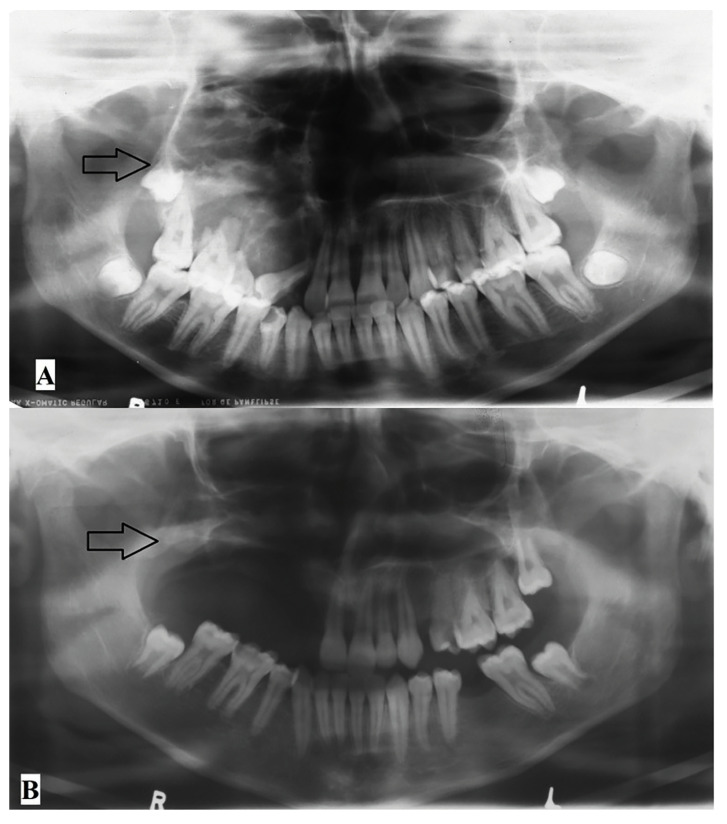
A: Pre-operative panoramic radiograph showing JTOF in the right posterior maxilla. B: Follow-up panoramic radiograph of the same patient three years post curettage with peripheral ostectomy showing no sign of recurrence of the lesion

Curettage in conjunction with peripheral ostectomy was shown to be the least invasive method with an accepTable recurrence rate of 10% (Fig. 4). Resection was performed in 6 patients with no recurrences however high morbidity. Statistically, no treatment modality was significantly less associated with recurrences than another method. A management protocol (Table 2) has been formulated based on these findings.

Overall, two recurrences occurred in this sample with a mean follow-up period of 20.5 months (recurrence rate: 11.8%). Both were of the JTOF variant. One lesion recurred in the posterior mandible while the other in the maxilla.

**Table 2:**
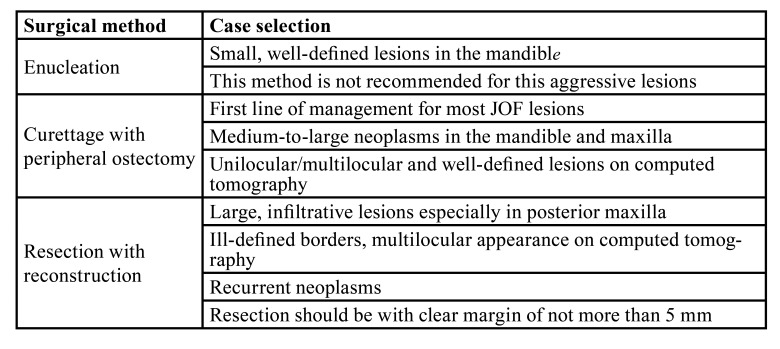
Protocol for surgical management of juvenile ossifying fibroma

## Discussion

JOF is a rare aggressive neoplasm with variable evidence regarding its diagnosis and management. Due to the similarities in the histopathological features of fibro-osseous lesions of the jaws, clinical, radiological and pathological correlations are needed to arrive at an accurate diagnosis (6). Both variants of JOF show some distinct histopathological features which make their diagnosis somewhat more predicTable, however correlating histopathological findings with clinical and radiological findings still forms an important part of the diagnostic process (2). Hence, this study aimed to present one of the largest cohorts of JOF in the literature and attempted to clarify some of the controversies surrounding this neoplasm. Moreover, this is the first study of its kind in a cohort of African patients with varying clinico-pathological features.

Age is an important factor in the diagnostic criteria of JOF. In 2005, the World Health Organization (WHO) reported the age of onset of JOF to be 15 years and younger (7,8); however, in the latest 2017 WHO classification no specific age category is mentioned (9). In a recent systematic review on JOF, Chrcanovic and Gomez (4) reported that the ages of patients with JPOF ranged between 0 - 68 years (mean= 18.9 years) while patients diagnosed with JTOF ranged between 1 - 33 years (mean= 11.5 years). This was found to be very similar in this sample and reaffirms that JOF predominately affects younger patients despite genetic and environmental differences.

In terms of gender distribution, a previous study by Han *et al*. (6) reported female predominance (M:F ratio= 1:2) however, Chrcanovic and Gomez (4) demonstrated an almost even distribution of JOF lesions amongst the sexes. Contrarily, males were more commonly affected in this African group of patients. Interestingly, all patients were of African and mixed race. These findings may be related to unique genetic factors that require further exploration.

Clinically, the majority of neoplasms caused rapid swelling in the maxillofacial region within a short period of time (4). This is one of the main reasons why the majority of patients seek medical opinion even though most lesions are painless as was shown in this series. The aggressive nature of this tumour is one of its defining criteria (1). Aggressive behaviour is measured by its rapid enlargement in a short period of time and its ability to invade or destroy adjacent anatomical structures. Most patients in this series showed signs of aggressive behaviour.

Site is another important factor in these neoplasms due to their ability to cause severe deformity of the facial skeleton. Most studies in the literature report that JTOF usually occurs in the maxilla while JPOF has a predilection for the para-nasal sinuses (4). Both lesions infrequently present in the mandible (4). Interestingly, the majority of lesions in this sample occurred in the mandible with only three JTOF neoplasms occurring in the maxilla and one in the sinonasal area. Even more surprising is the finding that all seven JPOF neoplasms occurred in the mandible. Williams *et al*. (10) also noted that 75% of lesions in their sample presented in the mandible. These differences indicate that perhaps site is not such an important factor in the clinico-pathological features of JOF as previously reported.

Radiological features of JOF form an important part of the clinico-pathological correlation process to reach an accurate diagnosis (1). A distinguishing feature of JOF and ossifying fibroma in general is that the neoplasm is well-demarcated and is of mixed radio-density (5). This aids to differentiate this lesion from fibrous dysplasia. The majority of lesions in this sample were well-defined and mixed in radio-density as was also reported by Chrcanovic and Gomez (4). Maxillary lesions were more likely to be ill-defined on radiographs.

More than three-quarters of JOF in the literature are reported to be unilocular in appearance (4). In this study, this was also found to be the case. Tooth displacement and root resorption were substantially more frequent in this sample than in the reported literature (4). The significantly larger size of JOF lesions when compared to conventional cement-ossifying fibromas in the jaws in the same population further shows the marked differences between these two variants of ossifying fibroma (11).

In terms of histopathological findings, JOF and specifically JPOF shows somewhat distinct features which aids in making an accurate diagnosis. Histopathological examination still forms the main diagnostic method in achieving a diagnosis. Most of these features are widely accepted in the literature. A debated topic is the association of aneurysmal bone cysts with JOF. This cohort of neoplasms showed similar incidence of aneurysmal bone cysts as in the literature (4).

Due to the aggressive nature of JOF and reported high recurrence rate, the best suited surgical method is widely debated. Han *et al*. (6) and Sarode *et al*. (12) advocated more radical treatment methods such as resection to manage these neoplasms. These radical options cause significant morbidity for these young patients with a lifetime of disFigurement albeit a very low recurrence rate. More recently, Chrcanovic and Gomez (4) in their systematic review reported accepTable results with curettage and peripheral ostectomy. This method has the advantage of reduced morbidity to the patient and has been recommended as the first line treatment for this lesion. Results from this study demonstrated similar findings in that curettage in conjunction with peripheral ostectomy had a low recurrence rate of 10%. Resection should be reserved for very aggressive or recurrent lesions while enucleation alone should be avoided as it has shown high recurrence (13). A management protocol has been proposed based on the findings in this study and supported by data from a recent systematic review by Chrcanovic and Gomez (4).

In conclusion, the high number of lesions occurring in the mandible for both variants of JOF demonstrates that site should not be a major determining factor in the diagnosis of JOF as previously reported. Moreover, curettage with peripheral ostectomy should be used as the first line of management to minimize morbidity to the patient and that resection should be reserved for very extensive and recurrent neoplasms.

## References

[1] El-Mofty S (2002). Psammomatoid and trabecular juvenile ossifying fibroma of the craniofacial skeleton: two distinct clinicopathologic entities. Oral Sur Oral Med Oral Pathol Oral Radiol Endod.

[2] Pimenta FJ, Gontijo Silveira LF, Tavares GC, Silva AC, Perdigão PF, Castro WH (2006). HRPT2 gene alterations in ossifying fibroma of the jaws. Oral Oncol.

[3] Slootweg PJ, Panders AK, Koopmans R, Nikkels PG (1994). Juvenile ossifying fibroma. An analysis of 33 cases with emphasis on histopathological aspects. J Oral Pathol Med.

[4] Chrcanovic BR, Gomez RS (2020). Juvenile ossifying fibroma of the jaws and paranasal sinuses: a systematic review of the cases reported in the literature. Int J Oral Maxillofac Surg.

[5] Noffke CE (1998). Juvenile ossifying fibroma of the mandible. An 8 year radiological follow-up. Dentomaxillofac Radiol.

[6] Han J, Hu L, Zhang C, Yang X, Tian Z, Wang Y (2016). Juvenile ossifying fibroma of the jaw: a retrospective study of 15 cases. Int J Oral Maxillofac Surg.

[7] Speight PM, Carlos R (2006). Maxillofacial fibro-osseous lesions. Diagn Histopathol.

[8] Slootweg PJ, Müller H (1990). Juvenile ossifying fibroma. Report of four cases. J Craniomaxillofac Surg.

[9] Speight PM, Takata T (2018). New tumour entities in the 4th edition of the World Health Organization Classification of Head and Neck tumours: odontogenic and maxillofacial bone tumours. Virchows Arch.

[10] Williams HK, Mangham C, Speight PM (2000). Juvenile ossifying fibroma. An analysis of eight cases and a comparison with other fibro-osseous lesions. J Oral Pathol Med.

[11] Titinchi F, Morkel J (2016). Ossifying fibroma: analysis of treatment methods and recurrence patterns. J Oral Maxillofac Surg.

[12] Sarode SC, Sarode GS, Waknis P, Patil A, Jashika M (2011). Juvenile psammomatoid ossifying fibroma: a review. Oral Oncol.

[13] Suarez-Soto A, Baquero-Ruiz de la Hermosa MC, Minguez-Martínez I, Floría-García LM, Barea-Gámiz J, Delhom-Valero J (2013). Management of fibro-osseous lesions of the craniofacial area. Presentation of 19 cases and review of the literature. Med Oral Patol Oral Cir Bucal.

